# Constitutive activation of CREB in mice enhances temporal association learning and increases hippocampal CA1 neuronal spine density and complexity

**DOI:** 10.1038/srep42528

**Published:** 2017-02-14

**Authors:** Tatsurou Serita, Hotaka Fukushima, Satoshi Kida

**Affiliations:** 1 Department of Bioscience, Faculty of Applied Bioscience, Tokyo University of Agriculture, 1-1-1 Sakuragaoka, Setagaya-ku, Tokyo 156-8502, Japan; 2 Core Research for Evolutionary Science and Technology (CREST), Japan Science and Technology Agency, Saitama 332-0012, Japan

## Abstract

Transcription factor CREB is believed to play essential roles in the formation of long-term memory (LTM), but not in learning and short-term memory (STM). Surprisingly, we previously showed that transgenic mice expressing a dominant active mutant of CREB (DIEDML) in the forebrain (DIEDML mice) demonstrated enhanced STM and LTM in hippocampal-dependent, rapid, one-trial learning tasks. Here we show that constitutive activation of CREB enhances hippocampal-dependent learning of temporal association in trace fear conditioning and delayed matching-to-place tasks. We then show that in DIEDML mice the apical tuft dendrites of hippocampal CA1 pyramidal neurons, required for temporal association learning, display increased spine density, especially of thin spines and of Homer1-negative spines. In contrast, the basal and apical oblique dendrites of CA1 neurons, required for rapid one-trial learning, show increased density of thin, stubby, and mushroom spines and of Homer1-positive spines. Furthermore, DIEDML mice showed increased dendritic complexity in the proximal portion of apical CA1 dendrites to the soma. In contrast, forebrain overexpression of CaMKIV, leading to enhanced LTM but not STM, show normal learning and CA1 neuron morphology. These findings suggest that dendritic region-specific morphological changes in CA1 neurons by constitutive activation of CREB may contribute to improved learning and STM.

Short-term memory (STM) is in a labile state up to several hours after learning and is stabilized through memory consolidation, thereby becoming long-term memory (LTM)^1^. Experiments in rodents using rapid one-trial learning paradigms such as Pavlovian fear conditioning showed that inhibiting gene expression blocks the formation of LTM without affecting STM^1^,^2^,^3^, indicating that consolidation of LTM depends on learning-induced gene expression, while STM does not.

The cAMP-responsive element-binding protein (CREB) is located downstream of the Ca^2+^ and cAMP signal transduction pathways that activate serine threonine kinase calcium/calmodulin-dependent protein kinase IV (CaMKIV) or protein kinase A (PKA), respectively^4^,^5^. Activated CaMKIV and PKA phosphorylate CREB at serine 133 (S133)^1^,^5^, and the S133-phosphorylated form of CREB (pCREB) then activates cAMP-responsive element (CRE) -mediated transcription^1^,^5^. Importantly, the loss of CREB or CaMKIV function disrupts memory consolidation; inhibition of CREB or CaMKIV function blocks the formation of LTM but does not impair STM^3^,^6^,^7^. Conversely, the gain of CREB function by transgenic expression of wild type (wt) CaMKIV or a constitutively active CREB mutant (CREB DIEDML or Y134F) in the forebrain enhances LTM formation^8^,^9^. Collectively, these findings indicate that the CaMKIV-CREB signaling pathway functions as a positive regulator of LTM consolidation.

Dendritic spines are small protrusions from neuronal dendrites that form the postsynaptic component of most excitatory synapses in the brain^10^. Dendritic morphology and patterning, including spine density and structure, help determine how input signals are processed in neural circuits, and are thought to reflect and/or determine connectivity between neurons at the synapse^11^,^12^,^13^. Importantly, dysfunctional dendritic morphology is often correlated with neurological disorders characterized by abnormalities in cognition, emotion, and memory loss^14^,^15^,^16^,^17^,^18^; reduced spine densities were observed in hippocampal neurons in mouse models of Alzheimer’s disease and in prefrontal cortical pyramidal neurons in subjects with schizophrenia^19^,^20^. Thus, dendritic morphology and patterning contribute to cognitive functions such as learning and memory as well as synaptic function.

Evidence was presented supporting a regulatory role of CREB in dendritic spine formation^21^. Blocking the CaMKIV-CREB pathway inhibits dendritic outgrowth^22^,^23^,^24^, whereas the acute expression of constitutively active CREB or wtCREB using a virus increased spine density in the hippocampal CA1 neurons of young adult rats and in principal neurons of the lateral amygdala in mice, respectively^25^,^26^,^27^. Importantly, up-regulation of CREB activity also leads to increased neuronal excitability^28^,^29^,^30^. Thus, it is possible that CREB plays roles in neural connectivity and/or activity by regulating dendritic morphology or number.

Interestingly, DIEDML and Y134F mice exhibited enhanced STM (30 min–2 h) in rapid, one-trial learning tasks^9^ that did not require learning-induced, CREB-mediated gene expression, raising the possibility that DIEDML mice show improved learning as well as STM processes. To test this possibility, we examined the effects of constitutive up-regulation of CREB activity on temporal association learning and on dendritic complexity and spine density in mouse hippocampal CA1 pyramidal neurons.

## Results

### CREB-DIEDML mice exhibit enhanced temporal association learning

Our previous study using transgenic mice expressing a dominant active CREB mutant (DIEDML or Y134F) showed that up-regulation of CREB activity enhanced long-term contextual fear, social recognition, and inhibitory avoidance memories that are generated following hippocampus-dependent rapid one-trial learning^9^. More interestingly, transgenic mice with up-regulated CREB activity also exhibited enhanced 30-min STMs that do not require learning-induced CREB-mediated transcription^9^. These observations raised the possibility that up-regulation of CREB improves even gene expression-independent learning processes. To test this hypothesis, we first performed a hippocampus-dependent, trace fear conditioning (TFC) temporal association learning task, where mice learned an association between a CS (tone) and a US (footshock) separated by a 30 s trace interval.

**Figure 1:**
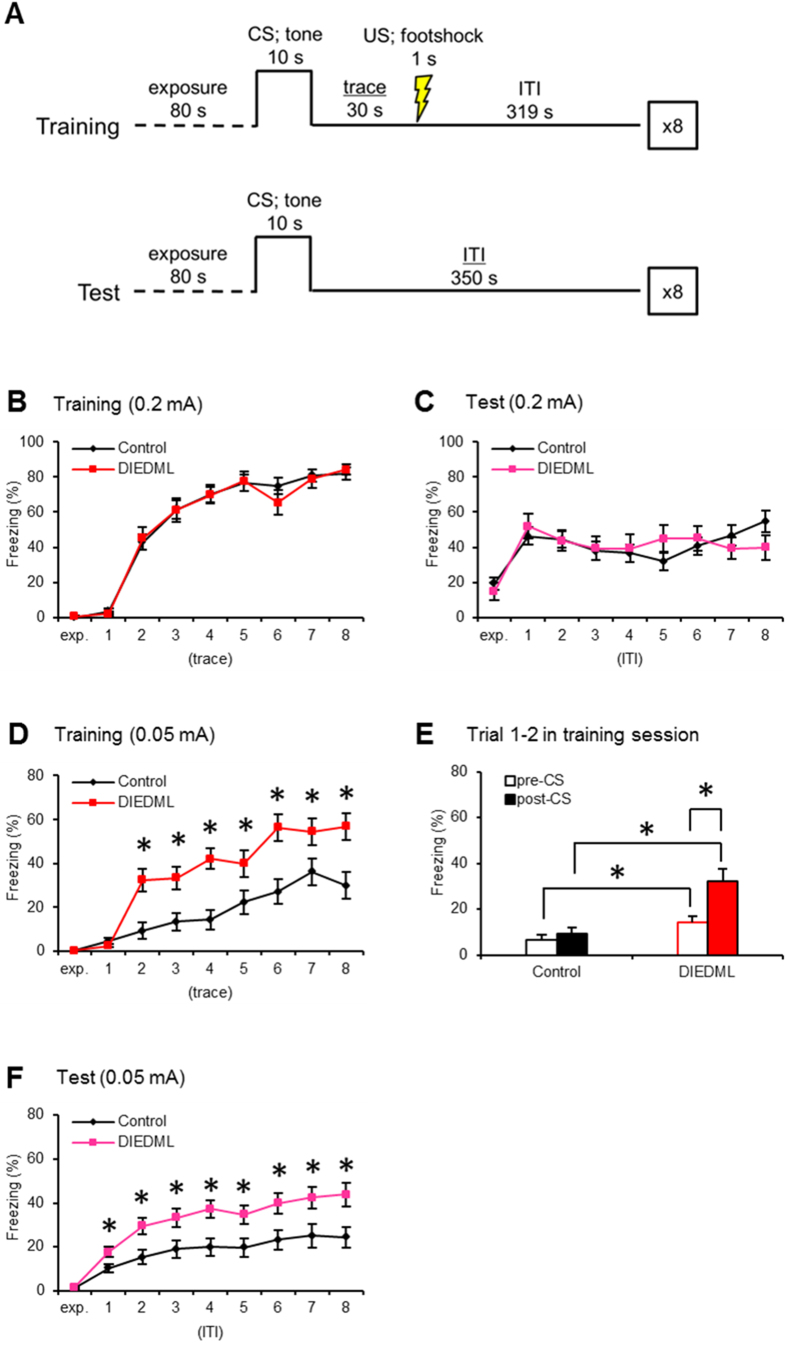
DIEDML mice display enhanced trace fear conditioning and subsequent memory. (**A**) Time course of trace fear conditioning training and testing. (**B**,**C**) Freezing responses during 0.2 mA footshock training (trace period) and test (ITI period) trials (control, n = 24; DIEDML, n = 17). (**D**,**E**) Freezing responses during 0.05 mA footshock training (trace period) and test (ITI period) trials (control, n = 26; DIEDML, n = 30). (**F**) Freezing levels during pre-CS (ITI of the Trial 1: 320 s) and post-CS period (trace of the Trial 2: 30 s) during the training session. CS; conditioned stimulus. US; unconditioned stimulus. ITI; inter-trial interval. *p < 0.05, compared with the control group. Error bars indicate SEM. The results of the statistical analyses are presented in Supplementary Table 1.

Both the DIEDML and WT mice exhibited similar freezing responses in the training and test trials when they were delivered 0.2 mA electrical footshocks (Fig. 1B,C; two-way repeated ANOVA, Supplementary Table 1). As it is possible that these strong training conditions enabled WT mice to learn and form a trace fear memory comparable to that of the DIEDML mice, we performed similar experiments using a weaker training protocol (0.05 mA electrical footshocks). In contrast to the results shown in Fig. 1B and C, two-way repeated ANOVA during training now revealed significant effects of trace, genotype, and trace × genotype interaction (Fig. 1D; Supplementary Table 1). DIEDML mice started to show significantly greater freezing response compared with WT mice immediately after they received the first trace fear conditioning during the training session (*post hoc* Bonferroni’s test, *ps* < 0.05). Importantly, two-way ANOVA comparing freezing of pre-CS (ITI of the Trial 1: 320 s) and post-CS (trace of Trial 2: 30 s) in the training session revealed significant effects of genotype, CS (pre VS post) and genotype × CS interaction (Fig. 1E; Supplementary Table 1). Most importantly, DIEDML mice (*p* < 0.05), but not WT mice (*p* > 0.05), showed significantly more freezing during post-CS compared with pre-CS and furthermore, showed significantly more freezing than WT mice (*p* < 0.05) (Fig. 1E). These observations suggest that DIEDML mice showed acquisition of trace fear conditioning at Trial 2 although WT mice failed to do. Additionally, paired *t* test confirmed this observation; only DIEDML mice showed significantly more post-CS freezing compared with pre-CS (*p* < 0.05). Taken together, these observations suggest that DIEDML mice learned the CS-US association significantly faster than WT mice.

Furthermore, DIEDML mice displayed a significantly greater freezing response compared with WT mice when they were re-exposed to the tones during the test trials (*ps* < 0.05), confirming our previous observations that DIEDML mice showed enhanced LTM (Fig. 1F; two-way repeated ANOVA, Supplementary Table 1). Thus our observations indicated that DIEDML mice showed enhanced learning of temporal association as well as formation of LTM.

**Figure 2:**
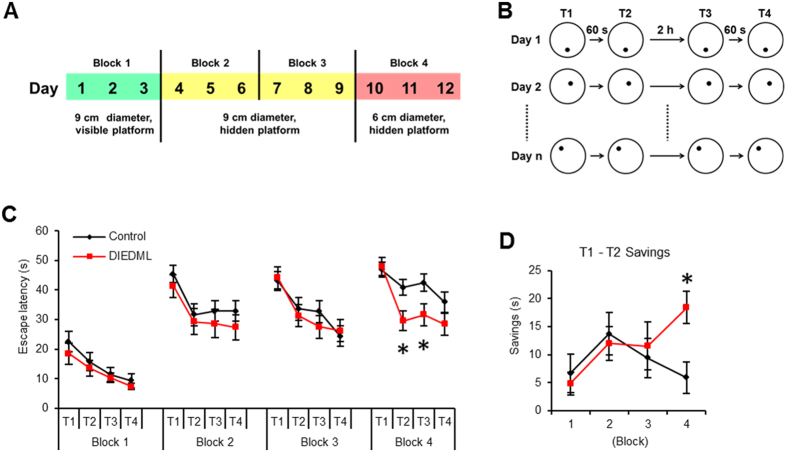
DIEDML mice display enhanced spatial working memory. (**A**,**B**) Time course of water maze version of the *delayed matching-to-place* task. (**C**) Averaged escape latencies for trial 1 (T1), T2, T3, and T4 for each 3-day block. (**D**) Averaged savings, differences in escape latency between Trial 1 and 2 (control, n = 13; DIEDML, n = 14). *p < 0.05, compared with the control group. Error bars indicate SEM. The results of the statistical analyses are presented in Supplementary Table 1.

To further examine the effects of up-regulated CREB activity on temporal association learning, we performed a water maze version of the delayed matching-to-place (DMP) task (Fig. 2A,B) to assess spatial working memory, another type of temporal association learning^31^. During Block 1 (platform visible, with flag), 2, and 3 (platform invisible, without flag), two-way repeated ANOVA revealed a significant effect of trial but not genotype and no trial × genotype interaction (Fig. 2C; Supplementary Table 1). DIEDML mice showed comparable escape latencies as WT mice during Blocks 1–3. In contrast, during Block 4 (smaller invisible platform), two- way repeated ANOVA revealed significant effects of trial, genotype, and trial × genotype interaction (Fig. 2C; Supplementary Table 1). DIEDML mice showed significantly shorter escape latencies to the platform during Trials 2 and 3 compared with WT mice (*ps* < 0.05), although these mutant mice displayed normal escape latency during Trial 1 (*p* > 0.05) (Fig. 2C). Consistently, DIEDML mice showed significantly more saving (differences of escape latency between Trial 1 and 2) at Block 4 compared with WT mice (Fig. 2D; unpaired *t* test, Supplementary Table 1). These results indicated that DIEDML mice showed enhanced spatial working memory. Collectively, our findings indicated that DIEDML mice display enhanced temporal association learning.

### Increased dendritic complexity of hippocampal CA1 pyramidal neurons in CREB-DIEDML mice

CA1 pyramidal neurons in the hippocampus receive input to their basal and apical oblique dendrites primarily from the CA3 area, and input to their apical tuft dendrites from the entorhinal cortex layer III (ECIII) and the thalamic nucleus reuniens^32^. Importantly, CA3-CA1 synaptic input is required for rapid one-trial contextual learning^33^,^34^, whereas ECIII-CA1 input is required for temporal association learning^35^. Thus CA1 neurons function as a center for receiving inputs from ECIII and CA3. Our previous and present studies demonstrate that DIEDML mice exhibit both enhanced hippocampus-dependent rapid one-trial memory and enhanced temporal association learning, raising the possibility that constitutive activation of CREB improves morphological signatures of neurons. Therefore, to examine this possibility, we analyzed the morphology of hippocampal CA1 pyramidal neurons.

**Figure 3:**
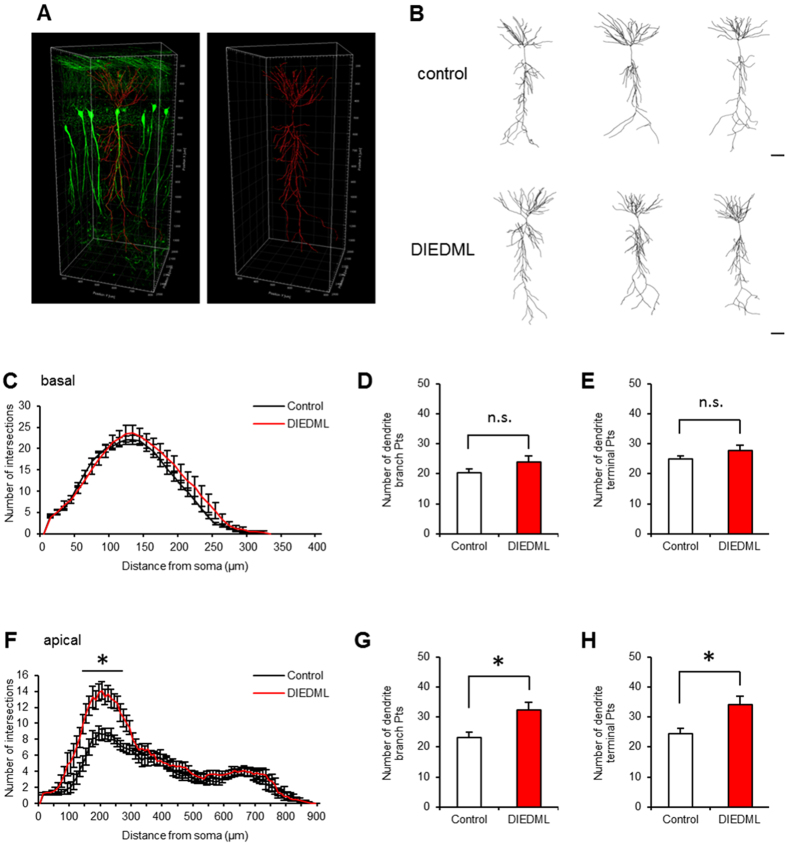
Increased dendritic complexity of hippocampal CA1 pyramidal neurons in DIEDML mice. (**A**) EGFP-labeled hippocampal CA1 pyramidal neurons visualized (green) and traced (red) in a CLARITY-processed tissue section. (**B**) Representative reconstructions of CA1 pyramidal neurons of control and DIEDML mice. Scale bar, 100 μm. (**C**) Sholl analysis of basal dendrites of hippocampal CA1 pyramidal neurons from DIEDML and control mice. (**D**,**E**) Quantification of the neuronal morphometric analysis, number of dendrite branch and terminal points of basal dendrites (n = 10 neurons per genotype, n = 5 mice per genotype). (**F**) Sholl analysis of apical dendrites of hippocampal CA1 pyramidal neurons from DIEDML and control mice. (**G**,**H**) Quantification of the neuronal morphometric analysis, number of dendrite branch and terminal points, at apical dendrites (n = 10 neurons per genotype, n = 5 mice per genotype). pts; points. *p < 0.05, compared with the control group. Error bars indicate SEM. The results of the statistical analyses are presented in Supplementary Table 1.

To image hippocampal neurons, CREB-DIEDML mice were crossed with Thy1-EGFP line M mice that express EGFP in sparse neurons^36^, generating double transgenic mice (Thy1/DIEDML Tg mice). 3D and 2D reconstructed neurons are shown in Fig. 3A and B, respectively. Sholl analyses showed that DIEDML mice exhibited dendritic complexity at the basal dendrites of their pyramidal neurons comparable to that of the control mice (Fig. 3C; two-way repeated ANOVA, Supplementary Table 1). Accordingly, DIEDML mice had comparable numbers of dendritic branch points and terminal points at the basal dendrites of CA1 neurons as the control mice (Fig. 3D,E; unpaired *t* test, Supplementary Table 1). By contrast, DIEDML mice exhibited an increase in dendritic complexity in the proximal portion of their apical CA1 dendrites (*ps* < 0.05) (Fig. 3F; two-way repeated ANOVA; Supplementary Table 1). DIEDML mice had significantly greater numbers of dendritic branch points and terminal points at the apical dendrites compared with control mice (Fig. 3G,H; unpaired *t* test; Supplementary Table 1). These results indicated that DIEDML mice display increased dendritic complexity only in the apical compartment of CA1 pyramidal neurons.

### Increased dendritic spine density of hippocampal CA1 pyramidal neurons in CREB-DIEDML mice

**Figure 4:**
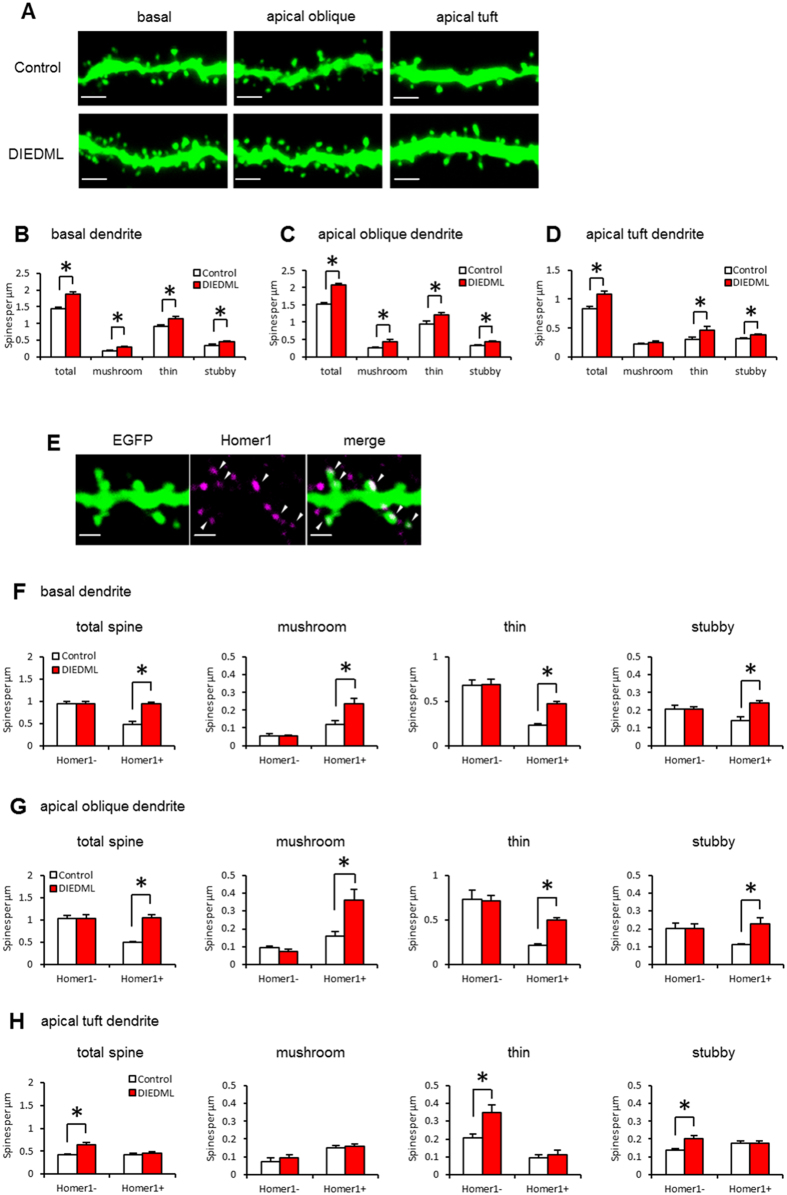
Increased dendritic spine densities on hippocampal CA1 pyramidal neurons in DIEDML mice. (**A**) Representative confocal images of basal, apical oblique, and apical tuft dendrites in CA1 pyramidal neurons of control and DIEDML mice. Scale bar, 2 μm. (**B**–**D**) Quantitative analysis of spine density and spine class (control n = 7 mice, n = 48–54 dendrites; DIEDML n = 6 mice, n = 40–47 dendrites). (**E**) Representative immunohistochemical staining of Homer1 (purple) in a CA1 pyramidal neuron dendrite. Arrow heads indicate Homer1-positive spines. Scale bar, 1 μm. (**F**–**H**) Quantitative analysis of Homer1-positive and -negative spine density and spine class (control n = 7 mice, n = 48–54 dendrites; DIEDML n = 6 mice, n = 40–47 dendrites). *p < 0.05, compared with the control group. Error bars indicate SEM. The results of the statistical analyses are presented in Supplementary Table 1.

We further analyzed dendritic spine morphology in the CA1 pyramidal neurons of DIEDML and control mice. Interestingly, DIEDML mice showed significant increases in the total spine density on basal, apical oblique, and apical tuft dendrites compared with control mice (Fig. 4A–D; unpaired *t* test, Supplementary Table 1). Classification of spine morphologies revealed significant increases in mushroom, thin, and stubby spines on basal and apical oblique dendrites in DIEDML mice compared with control mice (Fig. 4A–C; unpaired *t* test, Supplementary Table 1). By contrast, a significant increase in thin and stubby spines, but not in mushroom spines was observed on apical tuft dendrites of DIEDML compared with control mice (Fig. 4A,D; unpaired *t* test, Supplementary Table 1). These observations suggest that increases in spine density observed in the CA1 neurons of DIEDML mice were attributed to increases in thin spines on apical tuft dendrites and in all types of spines on basal and apical oblique dendrites.

To further characterize the molecular signatures of dendrites spines in DIEDML mice, we used immunohistochemistry to measure the number of spines positive for the Homer1 protein since Homer1 accumulates in the postsynaptic density (PSD), contributes to enlargement and stabilization of dendritic spines^37^ and have been used as a marker of mature spine^38^,^39^,^40^ (Fig. 4E). The numbers of Homer1-positive and -negative spines were counted in CA1 pyramidal neurons of Thy1/DIEDML and Thy1 mice. A significantly higher density of total Homer1-positive spines (*ps* < 0.05), but not total Homer1-negative spines (*ps* > 0.05), was observed in the basal and apical oblique dendrites of DIEDML mice than of control mice (Fig. 4F,G; two-way ANOVA, Supplementary Table 1). Similar to the results shown in Fig. 4B and C, classification of spine morphologies revealed significantly more Homer1-positive (*ps* < 0.05), but not Homer1-negative (*ps* > 0.05), mushroom, thin, and stubby spines in basal and apical oblique dendrites in DIEDML mice than in control mice (Fig. 4F,G; two-way ANOVA, Supplementary Table 1).

By contrast, the apical tuft dendrites of DIEDML CA1 neurons showed a significantly higher density of total Homer1-negative spines (*p* < 0.05), but not total Homer1-positive spines (*p* > 0.05) and especially, significantly more Homer1-negative thin and stubby spines, than did control mice (*ps* < 0.05) (Fig. 4H; two-way ANOVA, Supplementary Table 1).

These results suggested that the higher spine density in DIEDML hippocampal CA1 pyramidal neurons is attributed to the greater number of Homer1-positive spines (mushroom, thin, and stubby spines) in the basal and apical oblique dendrites, and to Homer1-negative thin and stubby spines in apical tuft dendrites.

### Temporal association learning and dendritic spine morphology of CaMKIV mice

Our previous study showed that transgenic mice overexpressing CaMKIV in their forebrains (CaMKIV mice) displayed behavioral phenotypes distinct from DIEDML mice; CaMKIV mice showed enhanced LTM but normal STM, while DIEDML mice showed enhancement of both LTM and STM^8^. Thus, we also examined the effects of CaMKIV-overexpression at the behavioral and neuron-morphological levels.

**Figure 5:**
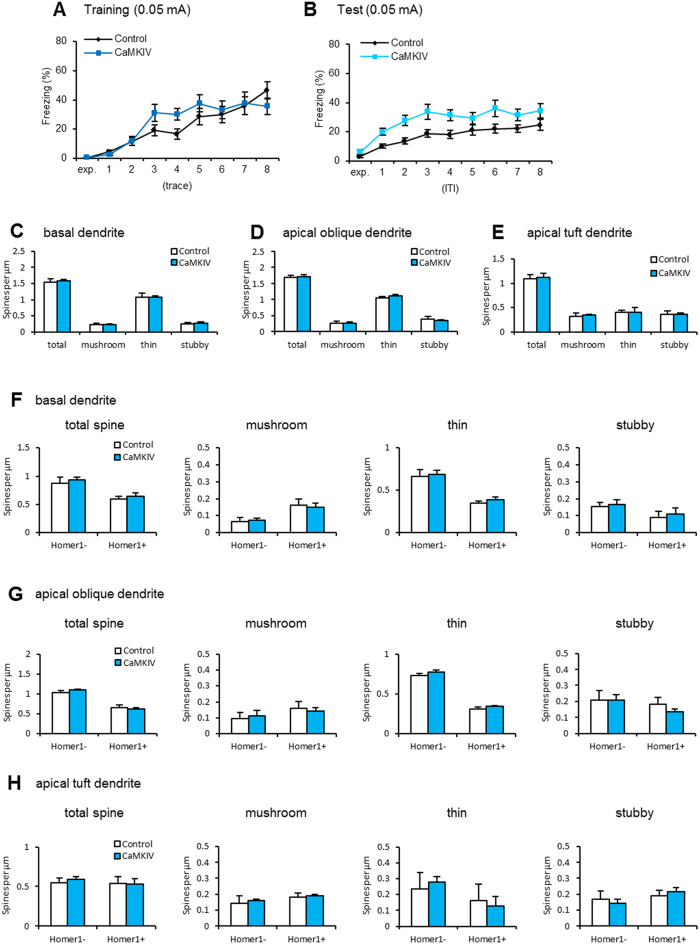
CaMKIV overexpression mice display normal temporal association learning and normal dendritic spine density. (**A**,**B**) Freezing responses during 0.05 mA footshock training (trace period; **A**) and test (ITI period; **B**) trials (control, n = 27; CaMKIV, n = 24). (**C**–**E**) Quantitative analysis of spine density and spine class (control n = 4 mice, n = 22–26 dendrites; CaMKIV n = 5 mice, n = 28–33 dendrites). (**F**–**H**) Quantitative analysis of Homer1-positive and Homer1-negative spine density and spine class (control n = 4 mice, n = 22–26 dendrites; CaMKIV n = 5 mice, n = 28–33 dendrites). Error bars indicate SEM. The results of the statistical analyses are presented in Supplementary Table 1.

We first examined the effects of up-regulated CaMKIV on temporal association memory using trace fear conditioning. We performed similar experiments to those shown in Fig. 1 using the weaker training conditions (0.05 mA) and CaMKIV mice. During training, two-way repeated ANOVA revealed significant effects of trace but not genotype or trace × genotype interaction (Supplementary Table 1). In contrast to the results from the DIEDML mice, CaMKIV mice showed normal temporal association learning (Fig. 5A). However, during the test trials, two-way repeated ANOVA revealed significant effects of trial and genotype but no trial × genotype interaction (Supplementary Table 1). CaMKIV mice displayed significantly larger freezing responses to the re-exposure to the tones compared with control mice (Fig. 5B; *p* < 0.05). Similar to previous observations^8^, these results indicated that CaMKIV mice show enhanced LTM but normal temporal association learning, confirming our previous observations.

We then analyzed the morphology of hippocampal CA1 neurons from CaMKIV mice. In contrast to the results from DIEDML mice, CaMKIV mice displayed a normal density of total spines as well as of the three types of spines (mushroom, thin, and stubby spines) on the basal, apical oblique, and apical tuft dendrites of hippocampal CA1 pyramidal neurons compared with control mice (Fig. 5C–E; unpaired *t* test, Supplementary Table 1). Additionally, no differences in the densities Homer1-positive and -negative spines were observed in CaMKIV and control mice (Fig. 5F–H; two-way ANOVA, Supplementary Table 1). These results indicated that the CaMKIV mice show normal morphology of hippocampal CA1 pyramidal neurons.

## Discussion

CREB was believed to play an essential role in the formation of LTM but not STM, by activating learning-induced transcription^3^,^6^. However, in a previous study of ours using rapid one-trial learning tasks, DIEDML mice exhibited enhanced STM as well as LTM^9^, raising the possibility that up-regulated CREB activity may enhance learning and the STM process. In this study, we tested this using temporal association learning tasks, and found that DIEDML mice learned the tone-and-shock association in trace fear conditioning and the location of the platform in a water maze delayed matching-to-place task significantly faster than WT mice (Fig. 2). Importantly, consistent with previous observations, DIEDML mice showed enhanced LTM in the trace fear conditioning task. These observations suggest that constitutive activation of CREB improves temporal association learning as well as LTM.

Temporal association learning and rapid one-trial learning differ in the circuit mechanisms. For instance, trace fear conditioning is dependent on hippocampus^41^,^42^,^43^ whereas delay (tone) fear conditioning is not (that depends on amygdala)^43^. More importantly, previous studies suggested that hippocampus-dependent temporal association learning requires ECIII-CA1 inputs to the apical tuft dendrites^35^, whereas hippocampus-dependent rapid one-trial learning (and spatial reference learning and memory in Morris water maze) requires CA3-CA1 synaptic inputs to the basal and apical oblique dendrites, of hippocampal CA1 neurons^33^,^34^,^44^. Thus CA1 neurons function as a center for temporal association and rapid one-trial learning by receiving inputs from ECIII and CA3, respectively. Our findings that DIEDML mice show improvement of both temporal association and rapid one-trial learning raised the possibility that constitutive activation of CREB alters the function of hippocampal CA1 neurons, thereby enabling improving learning and STM processes. Therefore, we compared the dendritic morphologies of hippocampal CA1 neurons in DIEDML and WT mice. Compared with WT mice, DIEDML mice had a higher density of thin spines throughout all of the dendritic sub-compartments and higher densities of mushroom and stubby spines only on the basal and apical oblique dendrites, but not on the apical tuft dendrites of hippocampal CA1 neurons. Additionally, the CA1 apical tuft dendrites in DIEDML mice had a higher density of Homer1-negative spines, while the basal and apical oblique dendrites had higher densities of Homer1-positive spines. DIEDML mice also exhibited increased dendritic complexity in the proximal portions of their dendrites. Together, these observations suggest that increased spine density and increased dendritic complexity on hippocampal CA1 neurons in DIEDML mice contribute to the enhanced rapid one trial and temporal association learning.

Thin spines emerge and disappear over a few days, and are therefore proposed to be flexible spines that are mainly known as “learning spines”^45^,^46^. By contrast, mushroom spines have larger PSDs^47^, which anchor more AMPA glutamate receptors and make these synapses functionally stronger^48^,^49^,^50^, and persist for months^51^. Therefore, mushroom spines are suggested to be stable “memory spines”^45^,^46^. Furthermore, previous studies suggested that dendritic spine density is associated with cognitive ability^46^,^52^. Interestingly, DIEDML mice had a higher density of thin spines on all of the sub-compartments of CA1 dendrites, and also had higher densities of mushroom and stubby spines on the basal and apical oblique dendrites. It is important to further clarify the roles of these increased densities of various spine types in learning, STM, and LTM in DIEDML mice. Additionally, it is possible that up-regulation of CREB activity also alters dynamics of spines such as formation and elimination. Therefore, further studies using *in vivo* imaging are required to examine relationships between spine dynamics and learning and memory in DIEDML mice.

The gene encoding brain-derived neurotrophic factor (*BDNF*) is a target of CREB^53^. Consist with this, DIEDML mice show an increase in *Bdnf* expression in the hippocampus^9^. There is abundant evidence that BDNF modulates dendritic complexity^54^,^55^,^56^,^57^ and the growth and formation of dendritic spines^57^,^58^. Dendritic complexity is increased in DG granule cells of transgenic mice overexpressing BDNF or in primary cultures of hippocampal neurons following BDNF application, and dendritic spine density is increased in primary or mature organotypic cultures of hippocampal neurons following BDNF application^56^,^57^,^58^. Importantly, the physiological variability in expression levels of BDNF in the mouse DG correlates with dendritic spine density in granule cells^59^. Taken together with these previous findings, our findings suggest that up-regulation of CREB increases spine density and complexity via increases in BDNF expression.

Importantly, hippocampal BDNF positively regulates STM and LTM in hippocampal-dependent one-trial learning tasks^60^. Consist with this, our previous study showed that the infusion of BDNF or a BDNF inhibitor (K252a) into the hippocampus of WT mice enhanced or impaired STM, respectively^9^. More importantly, we found that DIEDML mice with enhanced STM exhibited increased BDNF levels in the hippocampus, and that inhibiting hippocampal BDNF activity blocked this enhancement of STM, suggesting that up-regulation of CREB activity enhances STM via the increase in BDNF levels. Taken together with our findings that DIEDML mice show increased spine density and changes in spine morphology, suggest that up-regulation of CREB activity increases BDNF expression, thereby enhancing STM and learning via positive regulation of spine density and complexity.

In contrast to DIEDML mice, transgenic mice overexpressing CaMKIV in forebrain show normal STM^8^ in the rapid one-trial and temporal association learning tasks, but enhanced LTM in both tasks. Importantly, these transgenic mice showed normal hippocampal BDNF levels (data not shown) and spine morphology (Fig. 5). Therefore, these observations support our conclusion that up-regulation of CREB enhances learning and STM via changes in BDNF expression and spine morphology.

A previous study showed that viral expression of a constitutively active (ca) CaMKIV mutant in hippocampal CA1 neurons leads to an increase in spine density^25^. Importantly, in our study, we used mutant mice expressing wtCaMKIV, but not caCaMKIV in the forebrain. Taken together with our observation that these CaMKIV mutant mice show normal spine morphology and BDNF levels, we suggest that overexpression of wtCaMKIV in our mutant mice is insufficient for constitutive increase in CREB activity, thereby failing to improve STM and increase in spine density.

Previous studies have shown positive correlations between neuronal excitability and spine density^52^,^61^,^62^,^63^. Interestingly, the overexpression of wtCREB has been shown to increase neural excitability, thereby facilitating the allocation of these excited neurons into memory traces^29^,^64^. A recent study also suggests that neurons excited by the first learning are also easier to recruit into a memory trace at the second learning^30^. Therefore, it is possible that increases in spine density and dendritic complexity by up-regulated CREB activity contributes to increases in excitability and allocation of excited neurons into memory traces.

Temporal association learning was shown to depend on synaptic plasticity and metaplasticity of hippocampal CA1 neurons^41^,^65^. And it was recently shown that CA1-specific deletion of the metabotropic glutamate receptor 5 (mGluR5) required for metaplasticity of CA1 neurons results in impaired acquisition of trace fear conditioning^65^. We have just showed that DIEDML mice have altered numbers of Homer1-positive and -negative dendritic spines on CA1 neurons. It is possible that these changes in Homer1-positive and -negative spine densities modify synaptic strength, thereby enhancing temporal association learning, as Homer1 contributes to synaptic stability and plasticity by forming a complex with mGlu5 and Shank in the PSD^37^,^66^. However, we observed contrasting increases in the numbers of Homer1-positive and -negative spines in the basal and apical oblique dendrites and apical tuft dendrites, respectively, of CA1 neurons of DIEDML mice. Further study is required to understand the roles of these contrasting changes in Homer1-positive and -negative spines in learning.

In this study, DIEDML mice displayed enhanced temporal association learning and global and dendritic region-specific morphological changes in spines of the hippocampal CA1 pyramidal neurons, including increases in dendritic complexity and spine densities, suggesting that the enhanced learning by constitutive activation of CREB is attributed to dendritic morphological changes. Our findings also suggest a possible mechanism by which CREB improves learning by regulating dendritic morphology under basal conditions.

## Methods

### Animals

Mice were housed in cages of five or six, maintained on a 12 h light/dark schedule, and allowed *ad libitum* access to food and water in their home cages. All experiments were conducted during the light phase of the cycle in an illuminated testing room, according to the *Guide for the Care and Use of Laboratory Animals, Japan Neuroscience Society and Tokyo University of Agriculture*. All the animal experiments were approved by the *Animal Care and Use Committee of Tokyo University of Agriculture* (authorization number: 250006). All surgical procedures were performed under Nembutal anesthesia, and every effort was made to minimize suffering. All experiments were conducted blind to the treatment condition of the mouse. Animal behavior was recorded using a video camera. The generation and maintenance of DIEDML and CaMKIV mice was described previously^8^,^9^. Each experimental group contains equal number of male and female mice. Behavioral analyses were performed using transgenic mice and their WT littermates. Thy1-EGFP line M mice were crossed with heterozygous DIEDML or CaMKIV mice to produce double transgenic reporter mice^33^. Morphological analyses were performed using untrained transgenic mice and their Thy1-EGFP littermates.

### Trace Fear Conditioning

The trace fear conditioning was performed as described previously^67^. Mice were trained in a conditioning chamber (17.5 × 17.5 × 15 cm) fitted with a stainless steel grid floor through which footshocks could be delivered. The conditioned stimulus (CS) was a 65 dB white noise, delivered for 10 s, and the unconditioned stimulus (US) was a 0.05 or 0.2 mA footshock for 1 s. Mice were acclimated to shock chambers for 80 s, and then presented with eight CS-trace-US-ITI trials {training: trace of 30 s, inter-trial interval (ITI) of 320 s}. One day after training, mice were acclimated for 80 s and subjected to eight CS-ITI trials (test; ITI of 350 s) in a novel chamber to test for trace fear memory (Fig. 1A). Learning and memory was assessed by calculating the percentage of time spent freezing in trace (training) or ITI (test). Freezing behavior (defined as the complete lack of movement, except for respiration) was measured automatically as described previously^68^ (OHARA Pharmaceutical).

### A Delayed Matching-to-Place Task for Mice in Water Maze

The task protocol used was similar to that reported for rats^31^. Mice were given training with 4 trials (Trial 1–4) per day for 12 days. The single escape platform was located 1 cm below the surface of the water, on successive days, in 1 of 12 separate places within the pool. The locations of the platform were altered from one day to the next in a pseudo-random fashion, and the use of two locations in the same quadrant on 2 successive days was avoided. The ITIs between Trial 1 (T1) and T2, T3, and T4 were 60 s, and the ITIs between T2 and T3 was 2 h. On days 1–3 (Block 1), the experiment was performed with a 9 cm diameter platform marked by an attached flag (10 cm high). On days 4–6 and days 7–9 (Block 2 and Block 3), the flag was removed to hide the 9 cm diameter platform. On days 10–12 (Block 4), the experiment was performed with a 6 cm diameter hidden platform. Savings in latency between T1 and T2 were measured.

### Dendritic morphology

Brain tissue was optically cleared using the CLARITY protocol^69^ (http://clarityresourcecenter.org/) modified for “passive clearing”^70^. Briefly, mice were transcardially perfused with hydrogel solution. The brains were then removed, incubated with hydrogel solution at 4 °C for 48 h, and polymerized for 3 h at 37 °C, before 1-mm coronal sections were generated. Each section was clarified with clearing solution (200 mM boric acid, 4% SDS, pH 8.5) at 55 °C for 5 days. The cleared sections were washed with PBST (PBS plus 0.1% Triton X-100) and submerged in 80% glycerol at room temperature. Fluorescence images were acquired using a confocal microscope (TCS SP8; Leica, Wetzlar, Germany) equipped with a 20× NA 0.75 objective and LAS AF software (Leica). 3D modeling and dendritic morphological analysis, including sholl analysis^71^, were performed using the measurement tool of the Imaris software (Bitplane). Two neurons per mice were imaged (n = 10 neurons per genotype) (Fig. 3).

### Dendritic spine analysis

After anesthetization with intraperitoneal sodium pentobarbital (Somnopentyl, 50 mg/kg body weight; Kyouritu Seiyaku, Tokyo, Japan), mice were perfused with 4% paraformaldehyde. The brains were then removed, fixed overnight, transferred to 30% sucrose, and stored at −80 °C. Coronal sections were generated using a cryostat. Fluorescence images were acquired using a confocal microscope (TCS SP8 Leica, Wetzlar, Germany) equipped with a 63× NA 1.4 oil-immersion objective and LAS AF software (Leica). Single dendrite segment per neuron was imaged in each dendritic sub-compartments. Segments were imaged at 15 × zoom. Equal cutoff thresholds were applied to all slices. All confocal stacks were acquired at 512 × 512 pixel resolutions with a z-step of 0.5 μm. Settings for pinhole size and gain were optimized initially and remained constant throughout imaging to ensure images were digitized under consistent illumination. Confocal stacks were analyzed semi-automatically with Neuronstudio software (http://research.mssm.edu/cnic/)^72^. Spine density was calculated as the number of spines divided by dendritic segment length. Spines were classified as stubby if they had a head to neck diameter ratio less than 1.1. Thin spines were identified by a head to neck diameter ratio greater than 1.1 and a head maximum diameter less than 0.35 μm. Mushroom spines were identified by a head to neck diameter ratio greater than 1.1 and a head maximum diameter greater than 0.35 μm. For Homer1 staining, free floating sections were incubated with a polyclonal rabbit primary antibody for anti-Homer1 (1:500; Frontier Institute, Japan) in blocking solution overnight. The sections were washed with PBS and then incubated with Alexa Fluor 647-conjugated anti-rabbit secondary antibodies (1:500; Jackson Immuno Research Laboratories, USA) for 1 h in blocking solution (Fig. 4).

### Data analysis

Data were analyzed using analysis of the variance (ANOVA). Two-way or two-way repeated ANOVA followed by post hoc Bonferroni’s comparisons, respectively, were used to analyze the effects of genotypes, trace, ITI, CS (pre VS post), trial, distance from soma or Homer1 expression. Unpaired *t* tests were used to analyze the differences of effects of genotype on dendritic spines and savings in MWM. Paired *t* test was used to analyze the differences of freezing scores between pre- and post-CS. All values in the text and figure legends are means ± SEM. These statistical data were shown in Supplementary Table 1.

## Additional Information

**How to cite this article:** Serita, T. *et al*. Constitutive activation of CREB in mice enhances temporal association learning and increases hippocampal CA1 neuronal spine density and complexity. *Sci. Rep.* **7**, 42528; doi: 10.1038/srep42528 (2017).

**Publisher's note:** Springer Nature remains neutral with regard to jurisdictional claims in published maps and institutional affiliations.

## Supplementary Material

Supplementary Information
