# Supplementary material for: Constitutive activation of CREB in mice enhances temporal association learning and increases hippocampal CA1 neuronal spine density and complexity
Source: Sci Rep. 2017 Feb 14;7:42528. doi: 10.1038/srep42528 (PMC5307365; doi:10.1038/srep42528)
Supplement: Supplementary Information [file srep42528-s1.pdf]

Constitutive activation of CREB in mice enhances temporal association learning and increases hippocampal CA1 neuronal spine density and complexity

Tatsurou SERITA[1], Hotaka FUKUSHIMA[1,2], Satoshi KIDA[1,2]

1: Department of Bioscience, Faculty of Applied Bioscience, Tokyo University of Agriculture, 1-1-1 Sakuragaoka, Setagaya-ku, Tokyo 156-8502, Japan

2: Core Research for Evolutionary Science and Technology (CREST), Japan Science and Technology Agency, Saitama 332-0012, Japan

Correspondence should be addressed to Satoshi Kida, Department of Bioscience, Faculty of Applied Bioscience, Tokyo University of Agriculture, 1-1-1 Sakuragaoka, Setagaya-ku, Tokyo 156-8502. E-mail: kida@nodai.ac.jp

Supplemental Materials

Supplementary Table 1. The results of statistical analysis in Figure 1-5. The asterisks indicate a significant difference.

Figure	Sample size	Statistical test	Values
1B	control, n=24; DIEDML, n=17	2-way repeated measure ANOVA; factor1: trace factor2: genotype interaction: trace x genotype	F(8,312)=132.779, p<0.001* F(1,312)=0.067, p=0.797 F(8,312)=0.396, p=0.922
1C	〃	2-way repeated measure ANOVA; factor1: ITI factor2: genotype interaction: ITI x genotype	F(8,312)=8.218, p<0.001* F(1,312)<0.001, p=0.994 F(8,312)=1.539, p=0.1428
1D	control, n=26; DIEDML, n=30	2-way repeated measure ANOVA; factor1: trace factor2: genotype interaction: trace x genotype	F(8,432)=31.203, p<0.001* F(1,432)=18.71, p<0.001* F(8,432)=3.788, p<0.001*
1E	〃	2-way ANOVA; factor1: genotype factor2: CS interaction: genotype x CS	F(1,108)=20.823, p<0.001* F(1,108)=9.377, p=0.002* F(1,108)= 5.115, p=0.025*
1F	〃	2-way repeated measure ANOVA; factor1: ITI factor2: genotype interaction: ITI x genotype	F(8,432)=25.896, p<0.001* F(1,432)=11.57, p=0.001* F(8,432)=2.035, p=0.041*
2C; Block 1	control, n=13; DIEDML, n=14	2-way repeated measure ANOVA; factor1: trial factor2: genotype interaction: trial x genotype	F(3,237)=18.561, p<0.001* F(1,237)=0.24, p=0.868 F(3,273)=1.055, p=0.307
2C; Block 2	〃	2-way repeated measure ANOVA; factor1: trial factor2: genotype interaction: trial x genotype	F(3,237)=10.901, p<0.001* F(1,237)=0.108, p=0.955 F(3,273)=1.834, p=0.179
2C; Block 3	〃	2-way repeated measure ANOVA; factor1: trial factor2: genotype interaction: trial x genotype	F(3,237)=15.273, p<0.001* F(1,237)=0.372, p=0.543 F(3,273)=0.728, p=0.536
2C; Block 4	〃	2-way repeated measure ANOVA; factor1: trial factor2: genotype interaction: trial x genotype	F(3,237)=16.192, p<0.001* F(1,237)=7.302, p=0.008* F(3,273)=3.092, p=0.027*
2D; Block 1	〃	Unpaired t-test	t(79)=0.447, p=0.655
2D; Block 2	〃	Unpaired t-test	t(79)=0.354, p=0.724
2D; Block 3	〃	Unpaired t-test	t(79)=0.378, p=0.705
2D; Block 4	〃	Unpaired t-test	t(79)=3.018, p=0.003*
3C	n=10 neurons per genotype; 5 mice per genotype	2-way repeated measure ANOVA; factor1: distance factor2: genotype interaction: distance x genotype	F(33,528)=114.144, p<0.001* F(1,528)=0.6, p=0.449 F(33,528)=1.051, p=0.393
3D	〃	Unpaired t-test	t(16)=1.396, p=0.181
3E	〃	Unpaired t-test	t(16)=1.402, p=0.179
3F	〃	2-way repeated measure ANOVA; factor1: distance factor2: genotype interaction: distance x genotype	F(89,1424)=47.719, p<0.001* F(1,1424)=0.8627, p=0.009* F(89,1424)=4.123, p<0.001*
3G	〃	Unpaired t-test	t(16)=3.148, p=0.006*
3H	〃	Unpaired t-test	t(16)=3.173, p=0.005*

Figure	Sample size	Statistical test	Values
4B; total	control n=7 mice, n=48-54 dendrites; DIEDML n=6 mice, n=40-47 dendrites	Unpaired t-test	t (11)=5.22, p<0.001*
4B; mushroom	〃	Unpaired t-test	t (11)=3.002, p=0.012*
4B; thin	〃	Unpaired t-test	t (11)=2.778, p=0.017*
4B; stubby	〃	Unpaired t-test	t (11)=2.432, p=0.033*
4C; total	〃	Unpaired t-test	t (11)=8.11, p<0.001*
4C; mushroom	〃	Unpaired t-test	t (11)=2.795, p=0.017*
4C; thin	〃	Unpaired t-test	t (11)=2.376, p=0.036*
4C; stubby	〃	Unpaired t-test	t (11)=3.145, p=0.009*
4D; total	〃	Unpaired t-test	t (11)=4.88, p<0.001*
4D; mushroom	〃	Unpaired t-test	t (11)=1.299, p=0.22
4D; thin	〃	Unpaired t-test	t (11)=2.338, p=0.039*
4D; stubby	〃	Unpaired t-test	t (11)=2.946, p=0.013*
4F; total	〃	2-way ANOVA;<br>factor1: genotype<br>factor2: Homer1<br>interaction: genotype x Homer1	F(1,22)=23.151, p<0.001*<br>F(1,22)=23.038, p<0.001*<br>F(1,22)=21.366, p=0.001*
4F; mushroom	〃	2-way ANOVA;<br>factor1: genotype<br>factor2: Homer1<br>interaction: genotype x Homer1	F(1,22)=9.227, p=0.006*<br>F(1,22)=38.718, p<0.001*<br>F(1,22)=8.632, p=0.007*
4F; thin	〃	2-way ANOVA;<br>factor1: genotype<br>factor2: Homer1<br>interaction: genotype x Homer1	F(1,22)=6.958, p=0.015*<br>F(1,22)=51.21, p<0.001*<br>F(1,22)=6.011, p=0.022*
4F; stubby	〃	2-way ANOVA;<br>factor1: genotype<br>factor2: Homer1<br>interaction: genotype x Homer1	F(1,22)=7.663, p=0.11<br>F(1,22)=0.913, p=0.349<br>F(1,22)=8.21, p=0.009*
4G; total	〃	2-way ANOVA;<br>factor1: genotype<br>factor2: Homer1<br>interaction: genotype x Homer1	F(1,22)=19.176, p<0.001*<br>F(1,22)=16.464, p<0.001*<br>F(1,22)=18.644, p<0.001*
4G; mushroom	〃	2-way ANOVA;<br>factor1: genotype<br>factor2: Homer1<br>interaction: genotype x Homer1	F(1,22)=8.905, p=0.006*<br>F(1,22)=33.2, p<0.001*<br>F(1,22)=13.137, p=0.001*
4G; thin	〃	2-way ANOVA;<br>factor1: genotype<br>factor2: Homer1<br>interaction: genotype x Homer1	F(1,22)=4.785, p=0.0396*<br>F(1,22)=36.466, p<0.001*<br>F(1,22)=6.127, p=0.021*
4G; stubby	〃	2-way ANOVA;<br>factor1: genotype<br>factor2: Homer1<br>interaction: genotype x Homer1	F(1,22)=5.098, p=0.034*<br>F(1,22)=1.525, p=0.23<br>F(1,22)=5.265, p=0.031*
4H; total	〃	2-way ANOVA;<br>factor1: genotype<br>factor2: Homer1<br>interaction: genotype x Homer1	F(1,22) = 12.277, p = 0.002*<br>F(1,22) = 6.753, p = 0.016*<br>F(1,22) = 6.478, p = 0.018*
4H; mushroom	〃	2-way ANOVA;<br>factor1: genotype<br>factor2: Homer1<br>interaction: genotype x Homer1	F(1,22)=0.739, p=0.399<br>F(1,22)=14.158, p<0.001*<br>F(1,22)=0.143, p=0.708
4H; thin	〃	2-way ANOVA;<br>factor1: genotype<br>factor2: Homer1<br>interaction: genotype x Homer1	F(1,22)=7.967, p=0.009*<br>F(1,22)=38.474, p<0.001*<br>F(1,22)=4.788, p=0.039*
4H; stubby	〃	2-way ANOVA;<br>factor1: genotype<br>factor2: Homer1<br>interaction: genotype x Homer1	F(1,22)=5.729, p=0.025*<br>F(1,22)=0.156, p=0.696<br>F(1,22)=5.541, p=0.027*

Figure	Sample size	Statistical test	Values
5A	control, n=27; CaMKIV, n=24	2-way repeated measure ANOVA;<br>factor1: trace<br>factor2: genotype<br>interaction: trace x genotype	F(8,392)=24.725, p<0.001*<br>F(1,392)=0.699, p=0.407<br>F(8,392)=1.662, p=0.105
5B	〃	2-way repeated measure ANOVA;<br>factor1: ITI<br>factor2: genotype<br>interaction: ITI x genotype	F(8, 392)=17.798, p<0.001*<br>F(1,392)=11.077, p=0.001*<br>F(8,392)=1.043, p=0.402
5C; total	control n=4 mice, n=22-26 dendrites; CaMKIV n=5 mice, n=28-33 dendrites	Unpaired t-test	t(7)=0.259, p=0.802
5C; mushroom	〃	Unpaired t-test	t(7)=0.138, p=0.894
5C; thin	〃	Unpaired t-test	t(7)=0.5, p=0.961
5C; stubby	〃	Unpaired t-test	t(7)=0.467, p=0.654
5D; total	〃	Unpaired t-test	t(7)=0.314, p=0.762
5D; mushroom	〃	Unpaired t-test	t(7)=0.039, p=0.969
5D; thin	〃	Unpaired t-test	t(7)=1.312, p=0.23
5D; stubby	〃	Unpaired t-test	t(7)=0.632, p=0.547
5E; total	〃	Unpaired t-test	t(7)=0.244, p=0.813
5E; mushroom	〃	Unpaired t-test	t(7)=0.393, p=0.705
5E; thin	〃	Unpaired t-test	t(7)=0.057, p=0.955
5E; stubby	〃	Unpaired t-test	t(7)=0.055, p=0.957
5F; total	〃	2-way ANOVA;<br>factor1: genotype<br>factor2: Homer1<br>interaction: genotype x Homer1	F(1,14)=0.495, p=0.493<br>F(1,14)=16.666, p=0.001*<br>F(1,14)=0.002, p=0.961
5F; mushroom	〃	2-way ANOVA;<br>factor1: genotype<br>factor2: Homer1<br>interaction: genotype x Homer1	F(1,14)=0.02, p=0.89<br>F(1,14)=13.291, p=0.002*<br>F(1,14)=0.19, p=0.669
5F; thin	〃	2-way ANOVA;<br>factor1: genotype<br>factor2: Homer1<br>interaction: genotype x Homer1	F(1,14)=0.436, p=0.519<br>F(1,14)=36.083, p<0.001*<br>F(1,14)=0.004, p=0.948
5F; stubby	〃	2-way ANOVA;<br>factor1: genotype<br>factor2: Homer1<br>interaction: genotype x Homer1	F(1,14)=0.3, p=0.592<br>F(1,14)=3.426, p=0.085<br>F(1,14)=0.013, p=0.911
5G; total	〃	2-way ANOVA;<br>factor1: genotype<br>factor2: Homer1<br>interaction: genotype x Homer1	F(1,14)=0.122, p=0.732<br>F(1,14)=103.519, p<0.001*<br>F(1,14)=1.162, p=0.2993
5G; mushroom	〃	2-way ANOVA;<br>factor1: genotype<br>factor2: Homer1<br>interaction: genotype x Homer1	F(1,14)=0.003, p=0.958<br>F(1,14)=1.7, p=0.213<br>F(1,14)=0.24, p=0.632
5G; thin	〃	2-way ANOVA;<br>factor1: genotype<br>factor2: Homer1<br>interaction: genotype x Homer1	F(1,14)=2.114, p=0.168<br>F(1,14)=282.947, p<0.001*<br>F(1,14)=0.022, p=0.885
5G; stubby	〃	2-way ANOVA;<br>factor1: genotype<br>factor2: Homer1<br>interaction: genotype x Homer1	F(1,14)=0.377, p=0.549<br>F(1,14)=1.53, p=0.236<br>F(1,14)=0.399, p=0.537
5H; total	〃	2-way ANOVA;<br>factor1: genotype<br>factor2: Homer1<br>interaction: genotype x Homer1	F(1,14)=0.061, p=0.807<br>F(1,14)=0.32, p=0.58<br>F(1,14)=0.137, p=0.716
5H; mushroom	〃	2-way ANOVA;<br>factor1: genotype<br>factor2: Homer1<br>interaction: genotype x Homer1	F(1,14)=0.262, p=0.616<br>F(1,14)=2.06, p=0.173<br>F(1,14)=0.05, p=0.826
5H; thin	〃	2-way ANOVA;<br>factor1: genotype<br>factor2: Homer1<br>interaction: genotype x Homer1	F(1,14)=0.004, p=0.953<br>F(1,14)=4.327, p=0.056<br>F(1,14)=0.546, p=0.472
5H; stubby	〃	2-way ANOVA;<br>factor1: genotype<br>factor2: Homer1<br>interaction: genotype x Homer1	F(1,14)=0.004, p=0.949<br>F(1,14)=1.886, p=0.191<br>F(1,14)=0.531, p=0.478
